# The Role of Grass *MUTE* Orthologues During Stomatal Development

**DOI:** 10.3389/fpls.2020.00055

**Published:** 2020-02-11

**Authors:** Laura Serna

**Affiliations:** Facultad de Ciencias Ambientales y Bioquímica, Universidad de Castilla-La Mancha, Toledo, Spain

**Keywords:** grasses, MUTE, orthologues, polarization, stomata, subsidiary cells

## Abstract

Gas exchange between the plant and the atmosphere takes place through stomatal pores formed by paired guard cells. Grasses develop a unique stomatal structure that consists of two dumbbell-shaped guard cells flanked by lateral subsidiary cells. These structures confer a very efficient gas exchange capacity, which may have contributed to the evolutionary success of grasses. Recent works have identified orthologues of Arabidopsis *MUTE* in three grass species: *BdMUTE* in *Brachypodium distachyon*, *BZU2/ZmMUTE* in maize, and *OsMUTE* in rice. These genes induce the recruitment of subsidiary cells, and it appears to rely upon the ability of intercellular movement, from the guard mother cell to subsidiary mother cells, of the proteins encoded by them. Unexpectedly, this function of these grass *MUTE* genes contrasts with that of Arabidopsis *MUTE*, which promotes guard mother cell identity. These *MUTE* orthologues also appear to control guard mother cell fate progression, with the action of *BdMUTE* being less severe than those of *BZU2/ZmMUTE* and *OsMUTE.* The emerging picture unravels that grass *MUTE* genes have not only diverged, due to neo-functionalization, from Arabidopsis *MUTE*, but also among them.

## Introduction

Plants colonized land more than 400 million years ago (Edwards et al., 1998; Berry et al., 2010). One of the key innovations that enabled this to be possible was the development of a waxy cuticle to prevent water loss from the plant surface (Berry et al., 2010). The appearance of this impermeable layer coincides with the presence of stomatal pores, thus allowing the uptake of carbon dioxide to perform photosynthesis with a minimal loss of water vapor (Edwards et al., 1998; Berry et al., 2010). These microscopic innovations, bordered by a pair of kidney-shaped guard cells (GCs), are conserved across all land plants except liverworts and some mosses and hornworts (Chater et al., 2017; Renzaglia et al., 2017). Although to date no other structures has replaced the stoma, its shape, and its relationship with other epidermal cells have changed over time. Grasses, which develop a unique stomatal structure consisting of two dumbbell-shaped GCs flanked by two lateral subsidiary cells (SCs) (Stebbins and Shah, 1960; Rudall et al., 2017; Hepworth et al., 2018; Nunes et al., 2019), are a beautiful example of these changes. In addition, several works comparing stomatal responses between grasses and species with different stomatal morphology suggest that the stomatal complexes of grasses increase stomatal responsiveness with large and rapid GC movements (Franks and Farquhar, 2007; Bertolino et al., 2019 and references therein). Moreover, it has even been proposed that this developmental innovation has contributed, at least in part, to the extraordinary evolutionary success of this plant group (Kellogg, 2001; Hetherington and Woodward, 2003; Chen et al., 2017).

**Figure 1:**
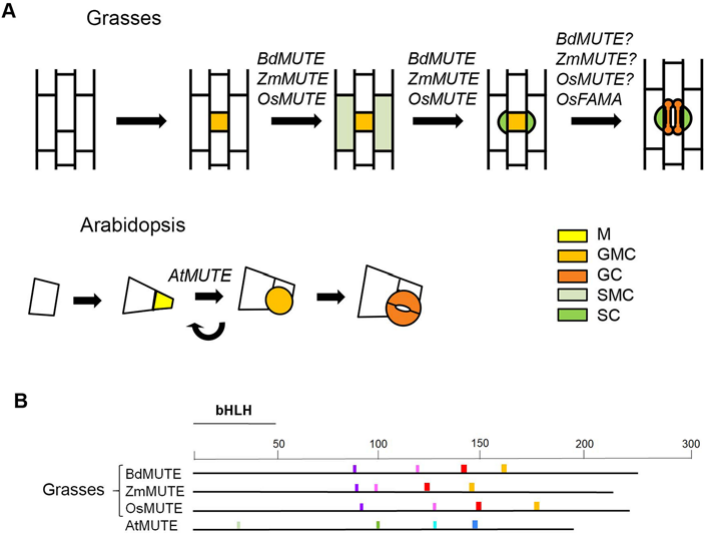
Role of *MUTE* orthologues of grasses and *AtMUTE* during stomatal development. **(A)** Stomatal development in grasses starts with an asymmetric division that produces a guard mother cell (GMC). Before GMC division, cells from files on either side of the GMC assume subsidiary mother cell (SMC) identity. SMCs then divide asymmetrically to produce subsidiary cells (SCs) in direct contact with the GMC. Only when the GMC is flanked by the two SCs, a symmetric cell division produces the two dumbbell-shaped guard cells (GCs). Grass *MUTE* genes specify SMC identity. They also appear to control the fate of the GMC, with the action of *BdMUTE* being less severe than those of *ZmMUTE* and *OsMUTE*. *OsFAMA* regulates the last stage of stomatal development. In Arabidopsis, a protodermal cell divides asymmetrically to produce a meristemiod (M) and a larger pavement cell. Ms usually reiterate asymmetric division several times, in an inward spiral, until they assume GMC identity. The GMC divides symmetrically to produce the two kidney-shaped GCs. *AtMUTE* regulates the transition of the M to GMC. **(B)** Schematic diagram of potential mobility motifs in the MUTE protein sequences. Conserved motifs in grass MUTE proteins could promote the intercellular movement, from the GMC to SMC, of these transcriptional factors. In contrast, the motifs conserved in AtMUTE could prevent its intercellular movement. The different-coloured boxes represent different motifs. These motifs are conserved among grasses but not in eudicots or vice versa, or are different between grasses and eudicots. Arabidopsis motifs are shown as an example of eudicot ones. The position of the bHLH domain is indicated. GC, guard cell; GMC, guard mother cell; M, meristemoid. SMC, subsidiary mother cell; SC, subsidiary cell.

In the leaves of grasses, stomatal development occurs only in some epidermal cell files and it proceeds acropetally, with early stages of this process taking place in the basal regions of the leaf and stomata developing later in the distal ones (Stebbins and Shah, 1960). The development of four-celled stomatal complexes takes place through a simple and invariant pattern of cell divisions (Stebbins and Shah, 1960; Serna, 2011; Hepworth et al., 2018; Nunes et al., 2019; **Figure 1A**). They initiate with an asymmetric cell division from a protodermal cells leading to a smaller guard mother cell (GMC) and a larger sister cell. Before GMC division, cells from files in either side of newly formed GMC acquire subsidiary mother cell (SMC) identity and divide asymmetrically. The smaller cells resulting from these divisions, which are always placed next to the GMC, differentiate as SCs. Following SCs recruitment, the GMC divides symmetrically, with the cell division plane being parallel to the main axis of leaf growth, and it yields the paired GCs. This cell division pattern differs from that taking place in Arabidopsis (Serna and Fenoll, 2000; Bergmann and Sack, 2007; **Figure 1A**). First, in Arabidopsis, stomatal precursors, named meristemoids, are self-renewing cells. They can undergo several rounds of cell division in an inward spiral, regenerating themselves in each division, before assuming GMC identity. In contrast, in grasses, an asymmetric division directly gives rise to the stomatal precursor. Thus, meristemoids appear to be absent in this plant group. Second, the GMC of Arabidopsis does not recruit SCs. In addition, while grasses form dumbbell-shaped GCs, eudicots and most monocots develop kidney-shaped GCs pairs (Stebbins and Shah, 1960).

**Figure 2:**
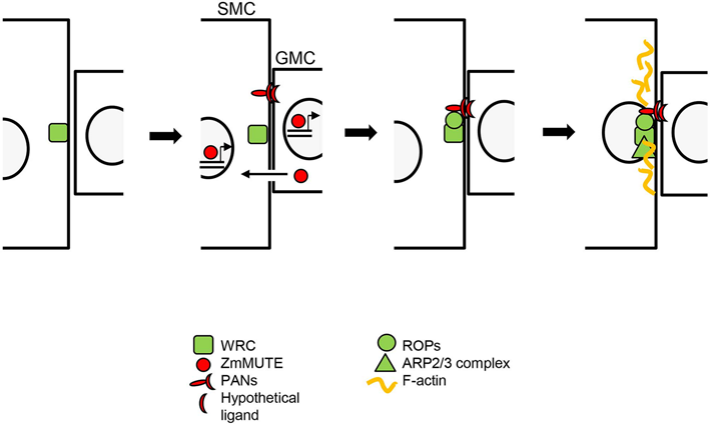
ZmMUTE role during the polarization of the subsidiary mother cell (SMC) in maize. The WAVE regulatory complex (WRC) complex polarizes to the cell surface of the SMC, at the site of guard mother cell (GMC) contact. ZmMUTE moves from the GMC to SMCs, where it binds to *PANs* promoters and promotes their induction. PANs accumulate, in a WRC-dependent manner, at the SMC/GMC contact site. PANs then recruit and activate ROPs. Activated ROPs physically interact and activate the WRC, which activates the ARP2/3 complex. Finally, ARP2/3 activity produces a dense F-actin patch and promotes nuclear migration towards the GMC in an actin-dependent manner. ZmMUTE may also exert a cell-autonomous role inducing, directly or indirectly, the expression of the hypothetical ligands of PANs. GMC, guard mother cell. SMC, subsidiary mother cell.

The recruitment of SCs in grasses is preceded by a process of polarization of the SMC that is very well known in maize (Serna, 2015; Apostolakos et al., 2018; **Figure 2**). This begins with the accumulation of the SCAR/WAVE regulatory complex (WRC) at the cell surface of the SMC, specifically at the site of GMC contact (Facette et al., 2015). Unknown signals emanating from GMC activate PANs receptors, which also, in a WRC-dependent manner (Facette et al., 2015), accumulate at the SMC/GMC contact site (Cartwright et al., 2009; Zhang et al., 2012). Then PANs recruit and activate ROPs (Humphries et al., 2011), which activate the WRC complex (Facette et al., 2015). Finally, activated WRC activates the ARP2/3 complex giving rise to a dense T-actin and inducing the migration of the nucleus toward the GMC (Deeks and Hussey, 2005).

Over the last twenty-five years, the isolation and characterization of numerous genes have illuminated our understanding of stomatal development in Arabidopsis. In this model plant, three basic helix-loop-helix (bHLH) transcription factors, SPEECHLESS (SPCH), MUTE, and FAMA sequentially specify stomatal lineage identity, regulate the transition from meristemoids to GMCs, and promote GCs differentiation, respectively (Ohashi-Ito and Bergmann, 2006; MacAlister et al., 2007; Pillitteri et al., 2007). The function of these transcriptional factors requires heterodimerization with the functionally redundant bHLH proteins ICE1 or SCREAM2 (Kanaoka et al., 2008). With origins which predate the divergence of the mosses and hornworts, these bHLH proteins diverged prior to the monocot-dicot split (Chater et al., 2017; Hepworth et al., 2018, and references therein). This divergence enabled the emergence of new protein functions, which are essential for the unique stomatal development of grasses (Raissig et al., 2017; Wang et al., 2019; Wu et al., 2019).

Recent works have uncovered the role of three orthologues of Arabidopsis *MUTE* during stomatal development in three grass species (Raissig et al., 2017; Wang et al., 2019; Wu et al., 2019): *BdMUTE* of *Brachypodium distachyon*, *BZU2/ZmMUTE* of *Zea mays* (maize) and *OsMUTE* of *Oryza sativa* (rice). The role of these genes, regulating the formation of SCs (Raissig et al., 2017; Wang et al., 2019; Wu et al., 2019), differs from that of *AtMUTE*. These orthologues of *MUTE* also seem to regulate the identity of the GMC, with the action of *BdMUTE* in this process being less severe. Taken together, these recent discoveries suggest not only that *MUTE* orthologues of grasses have diverged from *AtMUTE*, but also that *MUTE* genes of domesticated grasses studied thus far have diverged comparatively to *BdMUTE.*

## *MUTE* Orthologues of Grasses Recruit SCs

In *Brachypodium*, maize and rice, like in other grass species, stomatal complexes comprise a pair of dumbbell-shaped GCs associated with two SCs (Campbell, 1881; Stebbins and Shah, 1960; Shoemaker and Srivastava, 1973; Kamiya et al., 2003; Raissig et al., 2017). Interestingly, the mutants *subsidiary cell identity defective* (*sid*) of *B. distachyon*, *bizui2* (*bzu2*) of maize and *c-osmute* of rice, in addition to having impaired other steps of the stomatal developmental, fail to recruit SCs (Raissig et al., 2017; Wang et al., 2019; Wu et al., 2019). These mutants have alterations in their orthologues of Arabidopsis *MUTE* (Raissig et al., 2017; Wang et al., 2019; Wu et al., 2019): *sid* in *BdMUTE*, *bzu2-1* in *BZU2/ZmMUTE* and *c-osmute* in *OsMUTE.* The function of these *MUTE* orthologues contrasts with that of *AtMUTE*, which triggers GMC formation only in Arabidopsis (MacAlister et al., 2007; Pillitteri et al., 2007; **Table 1**).

**Table 1:** Functions of *AtMUTE* and *MUTE* orthologues of grasses.

Gene name	Species	Gene function	References
*AtMUTE*	*Arabidopsis thaliana* (Eudicot)	Transition from M to GMC	MacAlister et al., 2007; Pillitteri et al., 2007
*BdMUTE*	*Brachypodium distachyon* (Monocot, Poaceae)	Recruitment of SCs. Less severely, GMC and GCs identities	Raissig et al., 2017
*BZU2/ZmMUTE*	*Zea mays* (Monocot, Poaceae)	Recruitment of SCs and GMC identity. Early events in SMC polarization	Wang et al., 2019
*OsMUTE*	*Oryza sativa* (Monocot, Poaceae)	Recruitment of SCs and GMC identity	Wu et al., 2019

GCs, guard cells; GMC, guard mother cell; M, meristemoid; SMC, subsidiary mother cell; SCs, subsidiary cells.

How do these grass orthologues of Arabidopsis *MUTE* recruit SCs? The expression of the *yellow fluorescent protein* (*YFP*) under the control of the *BdMUTE* promoter showed that its induction is restricted to GMCs and GCs (Raissig et al., 2017). However, analysis of transgenic plants expressing the protein encoded by *YFP-BdMUTE* construct driven by the *BdSPCH2* promoter showed that BdMUTE locates not only in GMCs but also in SMCs (Raissig et al., 2017). Considering that the *BdSPCH2* promoter is active only in the stomatal lineage cells (Raissig et al., 2016), Raissig et al. (2017) inferred that BdMUTE protein moves from the GMC to epidermal cells of neighboring files. In consonance, successful complementation experiments of *sid* mutants with a fusion of the *BdMUTE* promoter to the *YFP-BdMUTE* construct lights up not only GMCs and young GCs, but also SMCs and young SCs (Raissig et al., 2017). In rice, *YFP-OsMUTE* expression driven by the *OsMUTE* promoter, whose induction in the developing four-celled complex is restricted to GMCs (Liu et al., 2009; Wang et al., 2019), lights up also GMCs and SMCs (Wang et al., 2019). This indicates that OsMUTE, like BdMUTE, also moves from the GMC to epidermal cells of neighboring files (Wang et al., 2019). Maize expressing *YFP-ZmMUTE* driven by the *ZmMUTE* promoter illuminates also GMCs, young GCs and SMCs (Wang et al., 2019). Assuming that the cellular localization of the *ZmMUTE* promoter induction is restricted to GMCs and young GCs, ZmMUTE would also move from the GMC to epidermal cells of neighboring files. Indeed, ZmMUTE protein is also able to move from the GMCs to SMCs in rice, and to epidermal cells adjacent to the stoma in Arabidopsis (Wang et al., 2019). These experiments strongly suggest that the recruitment of SCs in grasses depends on the intercellular movement of the grass MUTE proteins. Interestingly, the overexpression of *BdMUTE* driven by the *Ubi* constitutive promoter produces not only lateral, but also polar SCs (Raissig et al., 2017). This emphasizes the relationship between SCs recruitment and intercellular movement of MUTE orthologues in grasses. However, direct proof conclusively validating that the SCs formation relies upon the grass MUTE intercellular movement is lacking.

Multiple studies have shown that transcriptional factors move among cells *via* plasmodesmata (Han et al., 2014). GMCs of *Brachypodium* are symplastically connected with surrounding epidermal cells (Raissig et al., 2017). Therefore, BdMUTE may also move from the GMC to cells of neighboring files through plasmodesmata. But, what allows this protein to move laterally but not radially? Plasmodesmata continuously adjust their permeability in response to multiple cues (Sager and Lee, 2018). In addition, it is known that the control of this permeability is essential to the proper segregations of cell fate determinants during stomatal development in Arabidopsis (Guseman et al, 2010; Kong et al., 2012). Therefore, the lateral mobility of BdMUTE, and the unique design of the grass stomatal complexes, could depend on the restriction of the permeability of the plasmodesmata that symplastically connect cells of the same row. Future research should include delving in this direction.

## *ZmMUTE* Regulates Early Events in SMC Polarization

MUTE orthologues of grasses move from the GMC to the cells of neighboring files and this is linked with the formation of SCs. But, what do they do there? Wang and co-workers (2019) examined the role of *ZmMUTE,* specifically with regard to the regulation of SMC polarization. They found that cells adjacent to stomata, placed in neighboring epidermal files, of the *bzu2-1* mutant, in contrast to the wild type, do not show enrichment of F-actin patches at the GMC contact sites or polarization of their nuclei (Wang et al., 2019). This indicates that SMC polarization is not cell-autonomous, and that *ZmMUTE* regulates this process (**Table 1**). The *bzu2-1* mutation downregulates the transcription of both *PAN1* and *PAN2*, indicating that ZmMUTE transcriptional factor positively regulates *PAN1* and *PAN2* expression (Wang et al., 2019). Because PAN1, whose polarization at the SMC/GMC interface requires PAN2 (Cartwright et al., 2009; Zhang et al., 2012), recruits and activates ROPs (Humphries et al., 2011), ZmMUTE must induce *PANs* expression before ROPs polarization (**Figure 2**). Interestingly, while the *bzu2-1* mutant is almost devoid of SCs (Wang et al., 2019), most of the SCs of *pan1* and *pan2* mutants show no defects and most probably they derive from normal asymmetric cell divisions (Gallagher and Smith, 2000; Cartwright et al., 2009; Zhang et al., 2012; Facette et al., 2015). Therefore, ZmMUTE, or its downstream transcriptional factors, controls the expression not only of *PANs*, but also of other unknown genes to induce the recruitment of SCs. Among these genes could be those that encode for the hypothetical ligands of PANs, and his discovery would reveal one of the best-kept secrets of SMC polarization. If this is so, and assuming that these ligands emanate from the GMC, ZmMUTE may have both cell-autonomous and non-cell-autonomous functions (**Figure 2**).

Yeast one-hybrid and EMSA experiments showed that ZmMUTE binds to the E-box P1 and P2 motifs of the *PAN1* and *PAN2* promoters respectively (Wang et al., 2019). This suggests that the action of ZmMUTE on the expression of these genes may be direct. This does not rule out that ZmMUTE could also indirectly affect the activity of *PAN1* and *PAN2* promoters through upregulation of positive regulators and/or downregulation of repressors of *PANs* expression. For example, the transcriptional factor Glis3, directly and indirectly, regulates the expression of the insulin gene (Yang et al., 2009). The ZmMUTE protein does not bind the E-box P3 motif of the *PAN2* promoter *in vitro*, but ChlP-qPCR data indicate that it binds this motif *in vivo* (Wang et al., 2019). This suggests that the ZmMUTE protein physically interacts with other proteins to activate the E-box P3 of the *PAN2* promoter (Wang et al., 2019). Interestingly, yeast two-hybrid and bimolecular fluorescence complementation assays have just showed that its orthologue of rice, OsMUTE, interacts with OsICE1 and OsICE2 (Wu et al., 2019). Therefore, ZmMUTE may interact with their homologs to regulate the E-box P3 of the *PAN2* promoter.

## *AtMUTE* and Grass *MUTE* Orthologues Functions Have Diverged

BdMUTE, ZmMUTE and OsMUTE conserve the motifs that could promote their intercellular movement or lack those that could prevent such movement (Raissig et al., 2017; Xu et al., 2018; **Figure 1B**). These proteins move from the GMC to epidermal cells of neighboring files, where they may specify SMC identity to recruit SCs (Raissig et al., 2017; Wang et al., 2019; Wu et al., 2019). In contrast, the Arabidopsis MUTE protein, whose gene is expressed in GMCs (MacAlister et al., 2007; Pillitteri et al., 2007), does not move among cells (Wang et al., 2019). As expected, AtMUTE does not have the conserved mobility motifs of grass MUTE proteins, but those conserved in eudicots (Raissig et al., 2017; **Figure 1B**). In accordance, the recruitment of SCs does not take place in Arabidopsis. The role of AtMUTE is restricted to control the meristemoid to GMC transition (MacAlister et al., 2007; Pillitteri et al., 2007).

The fact that the *YFP-BdMUTE* construct, driven by the GMC-specific *AtMUTE* promoter (MacAlister et al., 2007; Pillitteri et al., 2007), illuminates not only stomatal precursors but also adjacent epidermal cells in Arabidopsis (Raissig et al., 2017; Wang et al., 2019), underlines the very likely importance of the mobility motifs in the protein movement. Interestingly, this construct does not induce the recruitment of SCs in Arabidopsis (Raissig et al., 2017; Wang et al., 2019), highlighting that *AtMUTE* and *BdMUTE* have diverged. *AtMUTEp : YFP-ZmMUTE* in Arabidopsis also lights up GMCs and neighboring epidermal cells (Wang et al., 2019). Given that OsMUTE conserve the motifs that could promote its movement or lack those that could prevent it (Raissig et al., 2017), it is expected that this protein also moves from stomatal precursors to neighboring epidermal cells in Arabidopsis. *OsMUTE* and *ZmMUTE* expressed under the control of the *AtMUTE* promoter partially complement the defects of Arabidopsis *mute-1* by inducing the formation of stomata from some stomatal precursors (Liu et al., 2009), but, like *BdMUTE*, they do not induce the recruitment of SCs (Figure 4 in Liu et al., 2009). Although OsMUTE and ZmMUTE, and perhaps BdMUTE, retain the function of inducing stomata formation, they are unable to induce the recruitment of SCs in Arabidopsis. This underlies that these proteins have diverged from the AtMUTE protein acquiring of a new function: the recruitment of SCs.

## *ZmMUTE* and *OsMUTE* Function Differs From That of *BdMUTE*

Although the three orthologues of *MUTE* regulate the formation of SCs, their function during stomatal development is not identical (**Table 1**). The *bzu2-1* mutant forms GMCs but displays defects in their divisions, undergoing excessive, randomly oriented and/or asymmetric divisions (Wang et al., 2019). This gives rise to short columns of elongated cells instead of stomata, which results in a slower transpiration rate and in a decreased photosynthetic activity (Wang et al., 2019). *c-osmute* exhibits also columns of undifferentiated cells, produced by misoriented and/or asymmetric cell divisions (Wu et al., 2019). Morphologically, the phenotype of the *c-osmute* mutant is reminiscent of the *bzu2-1* one, and the physiology of *c-osmute* mutants must also be dramatically affected. Certainly, both mutants exhibit a lethal phenotype at the seedling stage (Wang et al., 2019; Wu et al., 2019).

The *sid* mutant is fully viable and fertile, although its stomatal physiology is also affected (Raissig et al., 2017). Like the *bzu2-1* and *c-osmute* mutants, the *sid* mutant also undergoes misoriented GMC divisions (Raissig et al., 2017). However, in contrast to these mutants, about 70% of the GMCs of this mutant develop dicot-like two celled stomata (Raissig et al., 2017). Therefore, while *bzu2-1* and *c-osmute* mutants exhibit a fully penetrant phenotype affecting the division of the GMC (Wang et al., 2019; Wu et al., 2019), many of GMC divisions of the *sid* mutant are normal (Raissig et al., 2017). The molecular nature triggering the lack of a fully penetrant phenotype in *sid* is unknown, and to delve into this question is one of the most exciting future directions. *OsFAMA* also controls GC morphogenesis, with *c-osmute* exhibiting stomata with swollen GCs (Wu et al., 2019). This mutant also exhibits a fraction of swollen SCs, suggesting that, in addition to *OsFAMA*, other genes regulate SC differentiation (Wu et al., 2019). Among these unknown genes could be *OsMUTE*.

The defects induced by mutations in the grass *MUTE* orthologues in the maintenance of the GMC identity could reflect a mechanism of cellular signaling from the SMC towards the GMC to induce stomatal formation. It has been proposed that, prior SC formation, high levels of a grass peptide similar to AtEPF1/2 may cause GMC arrest (Hughes et al., 2017; Hepworth et al., 2018), perhaps through the suppression of grass MUTE orthologues activity specifically in GMCs. Grass MUTE activity in SMC would allow SC formation. Then, signals from SCs may activate grass MUTE orthologues in GMCs, perhaps by reducing grass EPF1/2 production, to promote stomatal formation. Agree with this, 1) GMCs do not progress to become stomata until SC formation, and 2) barley overexpressing *HvEPF1* exhibits arrested GMCs (Hughes et al., 2017). It is then likely that grass *MUTE* genes, in addition to having a non-cell-autonomous role specifying the SMC fate, have a cell-autonomous one triggering the progression of the GMC fate. The complementation of the Arabidopsis *mute-1* mutant phenotype, inducing stomatal development from some stomatal precursors, with at least the *OsMUTE* and *ZmMUTE* genes (Liu et al., 2009), also supports the cell-autonomous role of MUTE orthologues regulating GMC fate.

## Grass Stomatal Complexes Improve Stomatal Function

Several works comparing physiological stomatal behaviors among species with different stomatal complexes suggest that those of grasses are more efficient (Grantz and Zeiger, 1986; Franks and Farquhar, 2007; Vico et al., 2011; Merilo et al., 2014; McAusland et al., 2016; Haworth et al., 2018). The isolation of the *sid* mutant, the first grass mutant to date that disrupts the two main attributes of the grass stomatal complexes, the presence of dumbbell-shaped GCs and the recruitment of SCs (Raissig et al., 2017), underscores the important role of this innovative morphology in the stomatal function (Nunes et al., 2019). The maximum area of the open pore in the *sid* mutant, and its gas exchange capacity, were noticeably smaller than those in the wild type, even when stomatal opening was induced by the toxin fusicoccin (Raissig et al., 2017). The *sid* mutant also exhibited slower stomatal movements to fluctuating light conditions, and its stomata could not open as wide compared with the wild type (Raissig et al., 2017). Consequently, *sid* mutants produced less biomass than the wild types (Raissig et al., 2017). These results link the morphology of the stomatal complexes with its impact on gas exchange and biomass production in the wild grass *Brachypodium*, and strongly they suggest that this relationship may extend to the remaining grass species.

The improvement of stomatal function in grasses could have contributed to their expansion and diversification, 30 to 45 million years ago, when a progressive and global aridification took place (Kellogg, 2001; Hetherington and Woodward, 2003; Chen et al., 2017). The inability of the *sid* mutant to open widely its pores indicates that grass stomatal complexes are associated with greater stomatal openings and conductance. Interestingly, species with greater maximum stomatal conductance exhibit higher sensitivity to closure during drought (Henry et al., 2019). Under a global drought, a more sensitive stomatal closure could have allowed to capture carbon dioxide without losing too much water, thus favoring the successful diversification of this plant group. Certainly, Poaceae, with around 12,000 species, includes almost a quarter of all monocots of the planet, and it is one of the largest families of flowering plants. Curiously, the enrichment of species in genera of monocotyledons is associated with geographical variables, like larger ranges and lower elevations, rather than with biological attributes (Tang et al., 2016). It is likely that the success of the grasses lies partly in their morphology, including their unique stomatal complexes, and partly in the places they have occupied.

## Concluding Remarks

The development of the unique grass stomatal complex is a great advantage, which may have contributed to the expansion of this plant group. Undoubtedly, *MUTE* orthologues of grasses provide a starting point to unravel not only the mechanism underlying stomatal complexes formation, but also the evolution of this essential trait. *MUTE* orthologues of grasses have not only functionally diverged, due to neo-functionalization, from *AtMUTE*, but also among them, with *BdMUTE* exhibiting divergence from *ZmMUTE* and *OsMUTE*. Certainly, protein phylogenetic analysis of bHLH regulators of stomatal development supports this view (Wu et al., 2019). The comparison of the grass *MUTE* function between domesticated plants and their wild relatives, will allow us to know if the agricultural practices have driven the divergence of these genes. Because grass stomatal complexes have largely contributed to the adaptive success in hotter and drier environment, delving into the function of these genes will also provide useful genetic tools for producing plants with better tolerance to drought caused by climate change.

## Author Contributions

LS wrote the article and designed the figures.

## Conflict of Interest

The author declares that the research was conducted in the absence of any commercial or financial relationships that could be construed as a potential conflict of interest.
